# Supplementary material for: COVID-19 pandemic-related stress and substance use behaviors among people with HIV – a mixed method analysis
Source: PLoS One. 2026 May 28;21(5):e0349567. doi: 10.1371/journal.pone.0349567 (PMC13218522; doi:10.1371/journal.pone.0349567)
Supplement: Attachment — Submitted filename: PLOS review.docx [file pone.0349567.s002.docx]

This is a timely mixed-methods secondary analysis examining whether COVID-19–related stress and financial worry were associated with changes in opioid, cigarette, and alcohol use among people with HIV (PWH) with prior or current injection drug use in St. Petersburg, Russia, using data collected between May 2020 and July 2021. Key strengths include the focus on an under-studied, high-risk population outside the U.S. and the use of both survey and interview data to contextualize findings. With revisions—particularly clarifying measurement, outcome specification, statistical robustness, and interpretation—the paper could make a solid contribution.

**Introduction**

The introduction effectively highlights inconsistencies in prior findings. It would benefit from clearer justification of why PLWH are a particularly relevant population for studying pandemic stress and substance use, and how the Russian context may differ from other regions in shaping these experiences. Expanding on these points would better articulate the study’s contribution and research gap.

**Methods, Results, and Discussion**

Some details from the parent trials that are not directly analyzed here could be streamlined to avoid confusion (e.g., ART status, COVID infection history). The inclusion/exclusion table is useful, but the text should focus more tightly on variables relevant to this analysis.

The definition of “no change” combines “not changed,” “never used,” and “stopped using before the pandemic,” while “changed” includes increased, decreased, or stopped use due to the pandemic. Including never-users and pre-pandemic stoppers in the reference group may dilute or bias associations, particularly for alcohol and cigarettes, and creates inconsistencies across substances. If reanalysis is not feasible, a sensitivity analysis excluding these groups—or clearer discussion of this limitation—is recommended.

Causal language should be tightened. This is a cross-sectional analysis with stress and perceived change measured at the same time point, so directionality cannot be established. The manuscript should more clearly frame findings as associations and avoid implying causation.

Dichotomizing stress and financial worry at ≥4 on a 1–5 scale requires stronger justification or sensitivity analyses. Reporting correlations between stress and financial worry, or considering them jointly in models, would strengthen interpretation.

Interpretation of the main finding could be clearer. The outcome reflects “any change,” not direction, and most changes were decreases. Reframing conclusions accordingly would better align with the data. Language describing financial worry as “clinically meaningful” should be softened given imprecision in the estimate.

The mixed-methods approach appears more explanatory than truly convergent, as qualitative findings are used primarily for contextualization. Clarifying the design and noting reliance on English-language memos (rather than full transcript coding by the integrative analyst) as a limitation would improve transparency.

Selection bias is also possible, as only participants still engaged in follow-up completed the COVID survey. If available, describing differences between respondents and non-respondents—or acknowledging likely bias toward more stable participants—would be helpful.

**Other observations**

There is a time window inconsistency: the Abstract reports May 2020–March 2021, while the Methods reference interviews through July 2021. Please ensure consistent reporting.

The term “opioids” appears in qualitative narratives to include illicit opioids and methadone (and possibly diverted methadone). If possible, clarify what the opioid survey item specifically captured.

In Table 2, age is described as a mean in the text but presented as a median in the table; please make this consistent.

The four-category intervention variable combining study and arm should be clearly defined in the Methods, ideally with sample sizes per category.

Additional detail on qualitative rigor (e.g., whether saturation was assessed and how translation accuracy was ensured) would strengthen the qualitative methods.

While the Discussion appropriately emphasizes alignment with European findings, it would also be helpful to reiterate that this population—PWH with injection drug use in Russia—differs meaningfully from general population European surveys.
