# Supplementary material for: COVID-19 pandemic-related stress and substance use behaviors among people with HIV – a mixed method analysis
Source: PLoS One. 2026 May 28;21(5):e0349567. doi: 10.1371/journal.pone.0349567 (PMC13218522; doi:10.1371/journal.pone.0349567)
Supplement: Attachment — Submitted filename: Response to Reviewers.docx [file pone.0349567.s003.docx]

Dear Dr. Zimmerman,

Thank you for the opportunity to revise our manuscript entitled “COVID-19 pandemic-related stress and substance use behaviors among people with HIV – a mixed method analysis.” We appreciate the thoughtful and constructive feedback from you and the reviewer. We have revised the manuscript accordingly and believe these changes have strengthened the clarity and rigor of the paper. Below, we provide a detailed, point-by-point response to all comments. Changes in the manuscript are in tracked changes, and page numbers refer to the revised manuscript.

On behalf of all co-authors,

Sarah Rossi

**Editor’s Comments**

When submitting your revision, we need you to address these additional requirements.

**Editor comment 1:** Please ensure that your manuscript meets PLOS ONE's style requirements, including those for file naming. The PLOS ONE style templates can be found at

[https://journals.plos.org/plosone/s/file?id=wjVg/PLOSOne_formatting_sample_main_body.pdf](https://url.us.m.mimecastprotect.com/s/aG9JC68y2JCrBW8OZf2FlH5avlw?domain=journals.plos.org) and

[https://journals.plos.org/plosone/s/file?id=ba62/PLOSOne_formatting_sample_title_authors_affiliations.pdf](https://url.us.m.mimecastprotect.com/s/BtTWC82AOLS6KkLvBf9I5HyCANL?domain=track.editorialmanager.com)

**RESPONSE:** We have updated the formatting throughout the manuscript to align with PLOS ONE’s style requirements.

**______________________________________________________________________________**

**Editor comment 2:** Please include a complete copy of PLOS’ questionnaire on inclusivity in global research in your revised manuscript. Our policy for research in this area aims to improve transparency in the reporting of research performed outside of researchers’ own country or community. The policy applies to researchers who have travelled to a different country to conduct research, research with Indigenous populations or their lands, and research on cultural artefacts. The questionnaire can also be requested at the journal’s discretion for any other submissions, even if these conditions are not met. Please find more information on the policy and a link to download a blank copy of the questionnaire here: [https://journals.plos.org/plosone/s/best-practices-in-research-reporting](https://url.us.m.mimecastprotect.com/s/zt8WC9rBOMCkpO93rhqSqHqK64O?domain=journals.plos.org). Please upload a completed version of your questionnaire as Supporting Information when you resubmit your manuscript.

**RESPONSE:** We have uploaded a copy of PLOS’ questionnaire on inclusivity in global research and referenced it in the ethics statement in the methods section as a Supporting Information file.

**Changes in manuscript:** This questionnaire is referenced in the ethics statement on lines 310-312 and the caption to the supporting file is located on lines 926-928.

**______________________________________________________________________________**

**Editor comment 3:** We note that the grant information you provided in the ‘Funding Information’ and ‘Financial Disclosure’ sections do not match.

When you resubmit, please ensure that you provide the correct grant numbers for the awards you received for your study in the ‘Funding Information’ section.

**RESPONSE:** We have provided updated Financial Disclosure information in the revised cover letter to match the Funding Information section.

**______________________________________________________________________________**

**Editor comment 4:** Thank you for uploading your study's underlying data set. Unfortunately, the repository you have noted in your Data Availability statement does not qualify as an acceptable data repository according to PLOS's standards.

At this time, please upload the minimal data set necessary to replicate your study's findings to a stable, public repository (such as figshare or Dryad) and provide us with the relevant URLs, DOIs, or accession numbers that may be used to access these data. For a list of recommended repositories and additional information on PLOS standards for data deposition, please see [https://journals.plos.org/plosone/s/recommended-repositories](https://url.us.m.mimecastprotect.com/s/JGV9CgJK64fA7Bp04skUVH4TCzr?domain=track.editorialmanager.com).

**RESPONSE:** We have uploaded our minimal dataset to figshare. Please find the repository link here: <https://doi.org/10.6084/m9.figshare.31920138>

**______________________________________________________________________________**

**Editor comment 5:** Please include captions for your Supporting Information files at the end of your manuscript, and update any in-text citations to match accordingly. Please see our Supporting Information guidelines for more information: [http://journals.plos.org/plosone/s/supporting-information](https://url.us.m.mimecastprotect.com/s/x5vnCkRKD4COpw1WosYfGHGxA65?domain=track.editorialmanager.com).

**RESPONSE:** We have added captions for our Supporting Information file at the end of the manuscript.

**Changes in manuscript:** The caption for the supporting information file is on page 42, lines 925-928.

**______________________________________________________________________________**

**Editor comment 6:** We note that there is identifying data in the Supporting Information file < SCRIPT Combined IRB Docs and LINC-II IRB docs + translations>. Due to the inclusion of these potentially identifying data, we have removed this file from your file inventory. Prior to sharing human research participant data, authors should consult with an ethics committee to ensure data are shared in accordance with participant consent and all applicable local laws.

Data sharing should never compromise participant privacy. It is therefore not appropriate to publicly share personally identifiable data on human research participants. The following are examples of data that should not be shared:

-Name, initials, physical address
-Ages more specific than whole numbers
-Internet protocol (IP) address
-Specific dates (birth dates, death dates, examination dates, etc.)
-Contact information such as phone number or email address
-Location data
-ID numbers that seem specific (long numbers, include initials, titled “Hospital ID”) rather than random (small numbers in numerical order)

Data that are not directly identifying may also be inappropriate to share, as in combination they can become identifying. For example, data collected from a small group of participants, vulnerable populations, or private groups should not be shared if they involve indirect identifiers (such as sex, ethnicity, location, etc.) that may risk the identification of study participants.

Additional guidance on preparing raw data for publication can be found in our Data Policy ([https://journals.plos.org/plosone/s/data-availability#loc-human-research-participant-data-and-other-sensitive-data](https://url.us.m.mimecastprotect.com/s/XQ9MClYLD4U2vMpn6UvhLHzbkEI?domain=journals.plos.org)) and in the following article: [http://www.bmj.com/content/340/bmj.c181.long](https://url.us.m.mimecastprotect.com/s/DvqICmZ6G4HjrMDJksxiwHR4V2C?domain=bmj.com).

Please remove or anonymize all personal information (Name, Address and Dates), ensure that the data shared are in accordance with participant consent, and re-upload a fully anonymized data set. Please note that spreadsheet columns with personal information must be removed and not hidden as all hidden columns will appear in the published file.

**RESPONSE:** Thank you for highlighting this issue. After review, we have removed the Supporting Information file of qualitative quotes due to the sensitive nature of the qualitative data, the small sample size, and the potential risk of participant identification. In accordance with PLOS ONE’s data availability policy, we have made a de-identified quantitative dataset available via figshare (<https://doi.org/10.6084/m9.figshare.31920138>). Qualitative excerpts included in the manuscript have been carefully selected and reviewed to ensure that they do not contain identifying information.

**______________________________________________________________________________**

**Editor comment 7:** If the reviewer comments include a recommendation to cite specific previously published works, please review and evaluate these publications to determine whether they are relevant and should be cited. There is no requirement to cite these works unless the editor has indicated otherwise.

**RESPONSE:** Thank you for this clarification. The reviewer did not recommend citing specific previously published works.

**______________________________________________________________________________**

**Reviewers' comments:**

**Reviewer Comment 1:** This is a timely mixed-methods secondary analysis examining whether COVID-19–related stress and financial worry were associated with changes in opioid, cigarette, and alcohol use among people with HIV (PWH) with prior or current injection drug use in St. Petersburg, Russia, using data collected between May 2020 and July 2021. Key strengths include the focus on an under-studied, high-risk population outside the U.S. and the use of both survey and interview data to contextualize findings. With revisions—particularly clarifying measurement, outcome specification, statistical robustness, and interpretation—the paper could make a solid contribution.

**RESPONSE:** Thank you for your thoughtful review of our manuscript. We have made substantial revisions based on your feedback. Line numbers refer to the clean copy version.

**______________________________________________________________________________**

Introduction
**Reviewer Comment 2:** The introduction effectively highlights inconsistencies in prior findings. It would benefit from clearer justification of why PLWH are a particularly relevant population for studying pandemic stress and substance use, and how the Russian context may differ from other regions in shaping these experiences. Expanding on these points would better articulate the study’s contribution and research gap.

**RESPONSE:** We have added additional justification in the introduction on the relevance of PLWH and the Russian context in studying pandemic stress and substance use. Thank you for this suggestion.

**Changes in manuscript:** The additional justification has been added to the introduction on lines 113-122 and 138-152.

**______________________________________________________________________________**

Methods, Results, and Discussion
**Reviewer Comment 3:** Some details from the parent trials that are not directly analyzed here could be streamlined to avoid confusion (e.g., ART status, COVID infection history). The inclusion/exclusion table is useful, but the text should focus more tightly on variables relevant to this analysis.

**RESPONSE:** We have removed details about the parent studies that are not relevant to the present analysis.

**Changes in manuscript:** Removal of this information is on lines 173-181 and lines 213-215.

**______________________________________________________________________________**

**Reviewer Comment 4:** The definition of “no change” combines “not changed,” “never used,” and “stopped using before the pandemic,” while “changed” includes increased, decreased, or stopped use due to the pandemic. Including never-users and pre-pandemic stoppers in the reference group may dilute or bias associations, particularly for alcohol and cigarettes, and creates inconsistencies across substances. If reanalysis is not feasible, a sensitivity analysis excluding these groups—or clearer discussion of this limitation—is recommended.

**RESPONSE:** Thank you for this recommendation. We have reanalyzed the data per your suggestion. For each substance (opioids, cigarettes, alcohol), we reran the same adjusted main model but restricted the analytic sample to participants with pre-pandemic use of that substance. This approach removes participants that responded with “never used” or “stopped using before the pandemic.” This has impacted the results in tables 3-5 and the corresponding text in the methods and results. We also made additions to the descriptive table (Table 2), for more transparency on how the variables were constructed.

**Changes in manuscript:** Updates to the description of the variable are located on lines 236-250. Updates to the results are located in the abstract, Tables 2-5, and text on lines 314-383.

**______________________________________________________________________________**

**Reviewer Comment 5:** Causal language should be tightened. This is a cross-sectional analysis with stress and perceived change measured at the same time point, so directionality cannot be established. The manuscript should more clearly frame findings as associations and avoid implying causation.

**RESPONSE:** Thank you for pointing this out. We have updated language throughout the manuscript to frame the findings as associations and removed causal-implying language.

**Changes in manuscript**: Language updates occur throughout the manuscript, including pages have been made in lines 377-380, 513-515, 523-531, and 571-582.

**______________________________________________________________________________**

**Reviewer Comment 6:** Dichotomizing stress and financial worry at ≥4 on a 1–5 scale requires stronger justification or sensitivity analyses. Reporting correlations between stress and financial worry, or considering them jointly in models, would strengthen interpretation.

**RESPONSE:** We thank the reviewer for this suggestion. COVID-related stress and financial worry were moderately correlated when continuous (ρ=0.61) or dichotomized (Spearman ρ=0.41), indicating related but distinct constructs. We added this information in the results section. We conducted sensitivity models including both variables simultaneously, and we found that estimates were attenuated, likely reflecting shared variance and limited power. Importantly, results were consistent when modeling both variables as continuous predictors, with both stress and financial worry significantly associated with opioid use change. These findings support the robustness of our results and suggest a graded relationship rather than a strict threshold effect. These additional models are represented in tables 6 and 7 as sensitivity analyses.

**Changes in manuscript:** Correlation values are reported on lines 321-323. Additional sensitivity models reported in Tables 6 and 7, and lines 384-390.

**______________________________________________________________________________**

**Reviewer Comment 7:** Interpretation of the main finding could be clearer. The outcome reflects “any change,” not direction, and most changes were decreases. Reframing conclusions accordingly would better align with the data. Language describing financial worry as “clinically meaningful” should be softened given imprecision in the estimate.

**RESPONSE:** Thank you for this helpful comment. We have revised the Results and Discussion to more clearly reflect that the outcome represents any change in substance use rather than direction of change. Specifically, we now consistently describe findings as associations with “reporting any change” in opioid use. We also explicitly note in both the Results and Discussion that the majority of reported changes were decreases rather than increases, to better align interpretation with the observed data.

In addition, we have softened our interpretation of the association with financial worry. We have removed the phrase “clinically meaningful” and now describe this estimate as suggestive but imprecise, noting that the confidence interval includes the null.

**Changes in manuscript:** Language updates occur throughout the manuscript, including in lines 374-383, 509-516, 523-531, 572-582, and 610-618.

**______________________________________________________________________________**

**Reviewer Comment 8:** The mixed-methods approach appears more explanatory than truly convergent, as qualitative findings are used primarily for contextualization. Clarifying the design and noting reliance on English-language memos (rather than full transcript coding by the integrative analyst) as a limitation would improve transparency.

**RESPONSE:** We appreciate the reviewer’s comment regarding the explanatory role of qualitative data. While qualitative findings do provide important context for interpreting quantitative results, this study is best characterized as a convergent mixed-methods design, as both data types were collected concurrently and neither informed the collection of the other. We have revised the manuscript to reflect this terminology and added a citation to established mixed-methods frameworks. We have also added the memo process as a limitation.

Fetters MD, Curry LA, Creswell JW. [Achieving integration in mixed methods designs-principles and practices](https://pubmed.ncbi.nlm.nih.gov/24279835/). *Health Serv Res*. 2013;48(6 Pt 2):2134-2156. doi:10.1111/1475-6773.12117

**Changes in manuscript:** We have added a section on the mixed methods design on lines 199-206. The memo process limitation is noted in lines 594-599.

**______________________________________________________________________________**

**Reviewer Comment 9:** Selection bias is also possible, as only participants still engaged in follow-up completed the COVID survey. If available, describing differences between respondents and non-respondents—or acknowledging likely bias toward more stable participants—would be helpful.

**RESPONSE:** Thank you for pointing this limitation out. We have provided this context in the limitations section.

**Changes in manuscript:** Selection bias limitations are noted in lines 588-590.

**______________________________________________________________________________**

**Reviewer Comment 10:** Other observations
There is a time window inconsistency: the Abstract reports May 2020–March 2021, while the Methods reference interviews through July 2021. Please ensure consistent reporting.

**RESPONSE:** Thank you for catching this oversight. We have made the correction.

**Changes in manuscript:** The correction was made in line 56.

**______________________________________________________________________________**

**Reviewer Comment 11:** The term “opioids” appears in qualitative narratives to include illicit opioids and methadone (and possibly diverted methadone). If possible, clarify what the opioid survey item specifically captured.

**RESPONSE:** We thank the reviewer for this suggestion. The opioid survey item did not specify type of opioid and asked: “Has the coronavirus pandemic changed the amount or frequency of your opioid use?” We have clarified this in the methods section.

While the survey item did not distinguish between opioid types, prior literature indicates that illicitly manufactured methadone is the predominant opioid used in this setting. Consistent with this, all qualitative participants who specified their drug of choice reported using illicitly manufactured methadone. We have added this clarification in both the methods and the discussion.

**Changes in manuscript:** We clarified that the opioid survey item did not specify type of opioid and noted the predominance of illicit methadone use in this setting in lines 241-243 and 605-608.

**______________________________________________________________________________**

**Reviewer Comment 12:** In Table 2, age is described as a mean in the text but presented as a median in the table; please make this consistent.

**RESPONSE:** Thank you for this observation. We have made this correction, so the text describes the median.

**Changes in manuscript:** The type of measurement for age is updated on line 315.

**______________________________________________________________________________**

**Reviewer Comment 13:** The four-category intervention variable combining study and arm should be clearly defined in the Methods, ideally with sample sizes per category.

**RESPONSE:** Thank you for this suggestion. We have clarified the definition of the four-category intervention variable in the methods as the cross-classification of study (SCRIPT vs. LINC-II) and intervention assignment (control vs. intervention), and we now report the sample sizes for each category.

**Changes in manuscript:** Further detail on the definition of the intervention variable and sample sizes for each category are noted in lines 270-277.

**______________________________________________________________________________**

**Reviewer Comment 14:** Additional detail on qualitative rigor (e.g., whether saturation was assessed and how translation accuracy was ensured) would strengthen the qualitative methods.

**RESPONSE:** Thank you for this suggestion. We have added further detail under the qualitative data collection and qualitative analysis sub-sections of the methods section addressing qualitative rigor. First, we now explicitly describe our use of a range-maximizing sampling strategy, which was designed to capture diverse participant experiences rather than achieve thematic saturation. As such, thematic saturation was not the primary criterion guiding sample size, and we have clarified this distinction in the manuscript.

Second, we have added detail on translation procedures to enhance transparency. Interviews were conducted by a bilingual research team that included a native Russian speaker fluent in English and a native English speaker fluent in Russian. All translated excerpts included in the manuscript were reviewed and verified by both team members, with additional bilingual team members contributing to translation and analysis at multiple stages.

**Changes in manuscript:** Additional detail on qualitative rigor are noted in lines 218-225 and 284-304.

**______________________________________________________________________________**

**Reviewer Comment 15:** While the Discussion appropriately emphasizes alignment with European findings, it would also be helpful to reiterate that this population—PWH with injection drug use in Russia—differs meaningfully from general population European surveys.

**RESPONSE:** Thank you for this observation. We have added language to the Discussion to more explicitly highlight that our study population (people with HIV with a history of injection drug use in Russia) differs meaningfully from general population samples in European surveys. We now note key contextual factors, including structural vulnerability, stigma, and access to harm reduction services, which may shape both stress experiences and substance use behaviors and limit direct comparability.

**Changes in manuscript:** This clarification has been added to the Comparison with other studies section (lines 552-560).

**______________________________________________________________________________**
